# Visualizing the Role of Midwives in Planetary Health: A Proposed Graphic Model

**DOI:** 10.1111/jmwh.70159

**Published:** 2026-07-11

**Authors:** Monica Streater

**Affiliations:** ^1^ School of Nursing University of Minnesota Minneapolis Minnesota

**Keywords:** environmental health, midwifery practice, midwifery education, planetary health

## Abstract

Climate change and ecological degradation pose growing threats to public health, with profound consequences for birthing individuals and fetal and newborn outcomes. Planetary health provides a framework for understanding the interdependence between environmental systems and human well‐being, underscoring the responsibility of health care professionals to engage in sustainable and equitable care practices. Midwives, with their holistic, preventive, and relationship‐centered approach, are well suited to respond to these challenges. This article introduces a graphic model that illustrates how midwifery practice contributes to planetary health through the integration of ecological awareness, social justice, and community‐centered care. Informed by planetary health principles and midwifery philosophies and core competencies, the model highlights the profession's role in addressing interconnected environmental, social, and health challenges in a sustainable manner. Although attention to social and environmental determinants of health has long been central to midwifery, planetary health concepts have not yet been systematically embedded in midwifery education or clinical frameworks. The proposed model makes visible the ways planetary health is already reflected in midwifery practice while identifying opportunities for expansion, including attention to environmental exposures and advocacy for climate‐resilient care systems. By visually articulating the relationship between midwifery and planetary health, the model serves as a tool for reflection, education, and action, supporting midwives in advancing care grounded in ecological responsibility and social equity.

## INTRODUCTION

Planetary health is a solutions‐oriented, transdisciplinary field and social movement dedicated to understanding and addressing how human disruptions to Earth's natural systems impact human health and the health of all life on the planet.^1^ Although environmental health traditionally focuses on managing localized human exposures to toxins and pollutants, planetary health addresses the systemic interdependence between the longevity of human civilization and the stability of Earth's natural ecosystems. Although the integration of planetary health and sustainability principles into midwifery education and practice is critical, it remains limited. Predominant models of midwifery practice primarily focus on clinical factors, overlooking the complex interactions among social, environmental, and cultural determinants that influence health, including planetary health.

**QUICK POINTS**

✦ Midwives play a vital role in advancing planetary health through sustainable and equitable care.
✦ The graphic model visually connects midwifery practice with environmental and social determinants of health, providing a tool for education and action for midwives worldwide.
✦ Integrating planetary health into education prepares midwives to address climate‐related health challenges.

Advocacy for health equity, social justice, and ethical health policies has long been central to midwifery practice.^2^ Midwives play a vital public health role by ensuring access to clean water and sanitation during childbirth, promoting breastfeeding, supporting family planning, and encouraging behaviors, such as smoking cessation, during pregnancy all over the world.^3^, ^4^ Part of midwives’ expanded role has also become addressing reproductive justice, particularly amid global climate change. Midwives promote reproductive justice by advocating for regulatory and legislative changes that protect vulnerable populations from environmental toxins such as exposure to industrial chemicals and endocrine disruptors, which can play a significant role in the rising rates of adverse birth outcomes.^5^ This requires safeguarding everyone's right to give birth and raise children in safe, healthy environments. In the United States, recent political rollbacks on environmental protections, reproductive rights, and public health care funding threaten both maternal‐child and planetary health, undermining efforts to confront the intertwined crises of climate change, health care access, and social equity.^6^ As ecological crises mount, they increasingly jeopardize the foundations of public health, and, thus, there is a growing imperative to integrate planetary health advocacy into the national and international scope of midwifery practice.

## THE ECOLOGICAL CRISIS

The global ecological crisis, characterized by climate change, pollution, and biodiversity loss, constitutes a profound and escalating threat to maternal and child health worldwide, with the frequency and severity of related events continuing to rise.^7^ The World Health Organization (WHO) reports that 24% of global deaths stem from modifiable environmental factors.^8^ Although these crises impact all communities, climate change and environmental degradation are current realities that disproportionately worsen health inequities, particularly for vulnerable pregnant and birthing people.^3^, ^9^ Environmental toxins and endocrine‐disrupting chemicals significantly impair reproductive health by contributing to declining sperm quality and male infertility while also increasing the risk of adverse pregnancy outcomes such as spontaneous abortion, stillbirth, and preterm birth.^5^

To fully grasp the global scale and lived experience of this crisis, the International Confederation of Midwives (ICM) emphasizes the impact on communities and practitioners worldwide. The ICM's “Interlocked: Midwives and the Climate Crisis” report revealed that 75% of surveyed midwives reported that climate change is already harming the communities they serve.^10^ This harm manifests globally through rising rates of preterm births, increased food insecurity, and significantly restricted access to essential maternity care during disasters such as floods, wildfires, and extreme heat events. Climate change poses specific health risks to individuals who are pregnant, breastfeeding, and postpartum through factors such as extreme heat, poor air quality, and waterborne and foodborne illnesses.^11^ Climate hazards are globally linked to severe adverse pregnancy outcomes, including an increased risk of complications, low birth weight, and stillbirths.^5^, ^7^, ^12^ Furthermore, the American College of Nurse‐Midwives (ACNM) emphasizes the reproductive harms of environmental toxins, calling for greater awareness and prevention in care.^13^

The crisis also directly impacts the global midwifery workforce. Midwives are often the first and only health care providers on the ground, serving as critical first responders in climate crises. However, this dedication comes at a cost, with 76% of midwives reporting that the climate crisis has negatively impacted their work, leading to increased stress, burnout, and even displacement.^10^ As the climate crisis worsens, urgent, globally coordinated action to integrate planetary health principles into policy and practice is essential.^14^ The ICM report calls on governments and policymakers globally to integrate midwives into national climate response strategies and provide the necessary training and resources to strengthen their ability to respond to growing challenges.^10^ This includes calling for governments to include midwives in implementing mitigation strategies such as early warning systems and provider‐patient education on heat risks.^10^, ^15^ Importantly, midwifery care is inherently sustainable: by promoting physiologic birth, reducing unnecessary interventions, and providing care closer to home, midwives effectively lower the carbon footprint of health care services and support the creation of climate‐resilient health care systems.^10^

This article proposes a new graphic model designed by the author to illustrate the multifaceted and interconnected roles of midwives within the context of planetary health. The model conceptualizes midwifery practice within a broader ecological and social framework, emphasizing the inseparable links between maternal health and environmental sustainability. By framing midwifery through this visual and holistic lens, this article advocates for advancing midwifery education, practice, and policy toward a more integrated, planetary health–centered approach.

## SETTING PLANETARY HEALTH GOALS

In 2015, the United Nations developed a framework of 17 Sustainable Development Goals (SDGs) to address urgent global challenges such as poverty, inequality, climate change, environmental degradation, peace, and justice.^16^, ^17^ These goals emphasize a holistic approach that connects people, planet, and prosperity, aiming to promote health equity and environmental sustainability within health care systems.^16^, ^18^

The integration of ecological awareness into midwifery care models has since gained recognition in global health discussions.^19^ The Healthy People 2030 initiative prioritizes reducing environmental hazards by improving policy, infrastructure, and equitable access to clean air and water, which are all essential for healthier pregnancies and birth outcomes.^9^ Similarly, the WHO emphasizes that transitioning to midwifery models of care plays a vital role in promoting both maternal health and environmental sustainability.^20^ Saunders and Davis note that midwives are uniquely positioned to educate individuals on minimizing environmental exposures and to advocate for broader policies addressing the health consequences of climate change.^21^ This dual responsibility underscores the urgent need for education and clinical practice guidelines that equip midwives with the knowledge and tools to integrate environmental and maternal health considerations into their care.

At the national level, the ACNM acknowledges the serious health risks climate change presents.^5^, ^12^ The ACNM recently updated its *Core Competencies for Basic Midwifery Practice*, which focuses extensively on equity, diversity, social determinants of health, and public health but does not address climate change, environmental sustainability, or planetary health in any direct manner.^2^ ACNM's position statements on climate change recognize midwives as critical factors in educating patients, reducing health care's environmental footprint, and advocating for climate‐resilient and equitable health systems.^2^, ^12^ Furthermore, ACNM's position paper titled *The Global Health Competencies and Skills for Midwives* was developed to help educators and guide students by providing structure for evaluating available global education and experiential opportunities.^22^ Nine competencies were identified, with specific skills for each articulated. Although the document does not explicitly reference planetary health, the “Global Understanding” competency calls for midwives to understand the effects of environmental determinants, including climate change, water and air quality, sanitation, and food scarcity, on health across world regions, reflecting alignment with planetary health principles.^22^

The ICM has updated its midwifery essential competencies to include addressing climate change impacts on maternal and child health, highlighting the vital role midwives play in responding to climate‐related crises.^10^, ^23^ Furthermore, the ICM's report *Interlocked: Midwives and the Climate Crisis* calls for enhanced midwife training, resources, and inclusion in climate preparedness plans to strengthen their response capabilities.^10^

## INTEGRATING PLANETARY HEALTH INTO MIDWIFERY PRACTICE

Midwives provide care in diverse contexts, addressing not only the physiologic aspects of pregnancy and birth but also the broader social and structural factors that shape health outcomes. Notably, midwifery practice extends well beyond pregnancy care, and midwives offer essential sexual care, reproductive care, preventive care, menopausal care, and more to women and gender‐diverse individuals throughout their life span.^2^ Through this comprehensive approach to care, midwives play a vital role in promoting health equity, offering education, support, and advocacy in both clinical and community settings, especially where access to care is limited. For example, midwives may work with families with (or facing) socioeconomic challenges, individuals who have immigrated or migrated from another country, or those living in marginalized communities, and their care addresses not only physical health concerns but also the socioeconomic factors that contribute to their patients’ health disparities. These populations are also more disproportionally affected by environmental exposures.

Veidis et al calls for clinicians to serve as advocates for improving planetary health, stating “we can spur individual‐level behavior change and bottom‐up environmental action.”^13^ There are various roles clinicians play in promoting planetary health that include identifying pattens of illness in response to changing conditions, reducing health care waste, patient education, social and political activism, and education for future workforce.^14^ If we begin to view midwifery as inherently linked to planetary health, we can strengthen the ethical and long‐term foundation of the profession. This shift in perspective not only supports midwives but also enhances maternal health and planetary health outcomes by promoting care models that are community centered, low intervention, and ecologically mindful. Recent scholarship suggests that although some nurse educators and midwives have begun integrating planetary health concepts into education and clinical practice, these efforts remain emergent and fragmented, highlighting the need for broader systemic incorporation and curricular development.^24^

### Framework Synthesis

The 6 domains of the graphic model were developed through a narrative synthesis of foundational documents purposively selected for their global influence on midwifery and planetary health standards. More specifically, these standard‐setting documents have contributed complementary elements in sustainability and planetary health to the development of the proposed model. The primary documents reviewed included the *Planetary Health Education Framework* (PHEF),^25^ *ACNM Global Health Competencies and Skills for Midwives*,^22^ *ACNM Core Competencies*,^2^ *ICM Essential Competencies*,^23^ and the *United Nations Sustainable Development Goals*.^16^ This synthesis ensured that the resulting model remains grounded in established professional standards while bridging the gap between planetary health principles and midwifery practice. These domains reflect the overlapping priorities across clinical, education, and advocacy contexts in midwifery. Aligning these frameworks highlights the ways midwives can integrate advocacy, education, and evidence‐based practice to promote both planetary and human health in contemporary clinical and education settings (see Table 1). The PHEF, SDGs, ACNM Global Health Competencies and Skills for Midwives, and the ACNM Core Competencies and ICM Essential Competencies together offer a comprehensive, multilevel perspective on midwifery education and practice.^2^, ^18^, ^22^, ^23^, ^25^

**Table 1:** Foundational Frameworks and Their Contributions to the Planetary Health Graphic Model

Document or Framework	Contribution to the Proposed Model
PHEF^25^	Emphasizes systems thinking, environmental justice, and the deep interconnectedness of human and ecological health. It guides learners to recognize how environmental degradation, social inequities, and health outcomes are linked, fostering the skills needed to build sustainable, equitable solutions
SDGs^16^	Provides a globally recognized roadmap and framework for addressing interrelated global challenges through coordinated action across health, education, climate, and social systems to advance health equity, sustainability, and social responsibility
ACNM Global Health Competencies and Skills for Midwives^22^	Serves as an education guide referencing environmental determinants of health. Its specific inclusion of the “Global Understanding” competency reflects an emerging alignment with core planetary health principles
ACNM Core Competencies for Basic Midwifery Practice^2^	Ensures that the model remains fundamentally grounded in national, US midwifery–specific education standards, professional ethics, and person‐centered clinical care
ICM Essential Competencies for Midwifery Practice^23^	Grounds the framework in global midwifery–specific standards and ethics, specifically incorporating updated competencies (such as Competency 1p) for providing maternal‐newborn care during climate‐driven crises and natural disasters

Abbreviations: ACNM, American College of Nurse‐Midwives; ICM, International Confederation of Midwives; PHEF, Planetary Health Education Framework; SDG, Sustainable Development Goal.

### Mapping Planetary Health Domains in Midwifery

Integrating planetary health principles into midwifery requires aligning global frameworks, professional competencies, and the lived realities of education and practice. Table 2 maps the 6 domains of the proposed graphic model to these guiding frameworks, including the ACNM Core Competencies, ACNM Global Health Competencies and Skills for Midwives, ICM Essential Competencies, PHEF, and the United Nations SDGs. ^2^, ^18^, ^23^, ^25^ This mapping illustrates how each domain can be enacted in everyday education and clinical practice.

**Table 2:** Alignment of Planetary Health Model Domains With Global Midwifery Frameworks and Clinical Practice

Domain	Aligned Guiding Frameworks/Documents	Clinical and Education Relevance
Policy advocacy	ACNM Core Competencies: II. E and II. H PHEF: Movement Building and Systems Change ICM Core Competencies: 1. Professional Practice SDGs: 3, 13, 16, and 17 ACNM Global Health Competencies: Health System Strengthening and Global Understanding	Advocate for reproductive justice and equitable care Promote environmentally responsible health care systems Engage students in policy simulations and advocacy exercises Translate research into policy and programs Collaborate on sustainable programming and resource allocation
Ecological sustainability	ACNM Core Competencies: I. J and I. K PHEF: Interconnection within Nature and The Anthropocene and Health ICM Core Competencies: 1. Professional Practice SDGs: 6 and 12 ACNM Global Health Competencies: Global Understanding	Integrate sustainable practices in clinical care Advise clients on local environmental risk exposures (eg, heat, air quality) and mitigation strategies Model eco‐conscious behaviors Embed sustainability in simulation, curricula, and QI projects Identify how environmental factors (clean water, air, food scarcity) vary across world regions
Reproductive justice	ACNM Core Competencies: I. A and II. H PHEF: Equity and Social Justice ICM Core Competencies: 2. Core Midwifery Care SDGs: 3 and 5 ACNM Global Health Competencies: Health Equity and Justice and Professionalism/Ethics	Uphold bodily autonomy and climate‐resilient care Reflect on structural inequities and oppression Advocate for marginalized populations Engage marginalized populations in health care decision‐making Apply international standards for the protection of human subjects
Social determinants of health	ACNM Core Competencies: I. K, I. L, and II. F PHEF: Systems Thinking / Complexity‐based Approaches ICM Core Competencies: 3. Health Promotion and Community Development SDGs: 1 and 10 ACNM Global Health Competencies: Clinical Practice and Health Equity and Justice	Assess socioeconomic and environmental factors Connect clients to community resources Use case‐based learning, highlighting structural drivers of inequity Assess health issues of vulnerable populations (immigrants, refugees, displaced persons) Analyze how political and health care systems workforce issues determine population health
Environmental justice	ACNM Core Competencies: I. L and II. H PHEF: Equity and Social Justice ICM Core Competencies: 1. Professional Practice SDGs: 11 and 13 ACNM Global Health Competencies: Global Understanding and Research/CQI	Advocate for communities affected by environmental harms Recognize risk patterns and support local resilience Integrate advocacy into continuing education Mitigate the health effects of migration and travel Identify innovative solutions for global health problems caused by environmental stressors
Education and empowerment	ACNM Core Competencies: I. I and I. A PHEF: Movement Building and Systems Change ICM Core Competencies: 1. Professional Practice SDGs: 4 ACNM Global Health Competencies: Teaching/Learning, Communication, and Research/CQI	Foster ecological awareness and climate literacy Empower learners and clients Promote interprofessional collaboration and equity outcomes Use technology and translators to bridge communication gaps Foster lifelong learning and self‐evaluation

Abbreviations: ACNM, American College of Nurse‐Midwives; ACNM Core Competencies, ACNM Core Competencies for Basic Midwifery Practice^2^; ACNM Global Health Competencies, ACNM Global Health Competencies and Skills^2^ ^0^; CQI, continuous quality improvement; ICM, International Confederation of Midwives; ICM Core Competencies, ICM Essential Competencies for Midwifery Practice^2^ ^1^; PHEF, Planetary Health Education Framework^2^ ^5^; QI, quality improvement; SDG, Sustainable Development Goal.^1^ ^6^

## PROPOSED GRAPHIC MODEL

The purpose of this visual model is to illustrate how midwives contribute to planetary health by integrating ecological awareness, social justice, and community‐centered care into their practice (Figure 1). The model aims to highlight midwifery's unique role in addressing interconnected health, environmental, and social challenges in a sustainable and equitable way. The model emphasizes midwives’ unique capacity to reduce health care's environmental footprint, educate patients on exposures, and promote sustainable, equitable systems of care. By visualizing these connections, the model serves as a tool for education, advocacy, and practice transformation, thus positioning midwives as essential partners in shaping a healthier planet for future generations.

**Figure 1:**
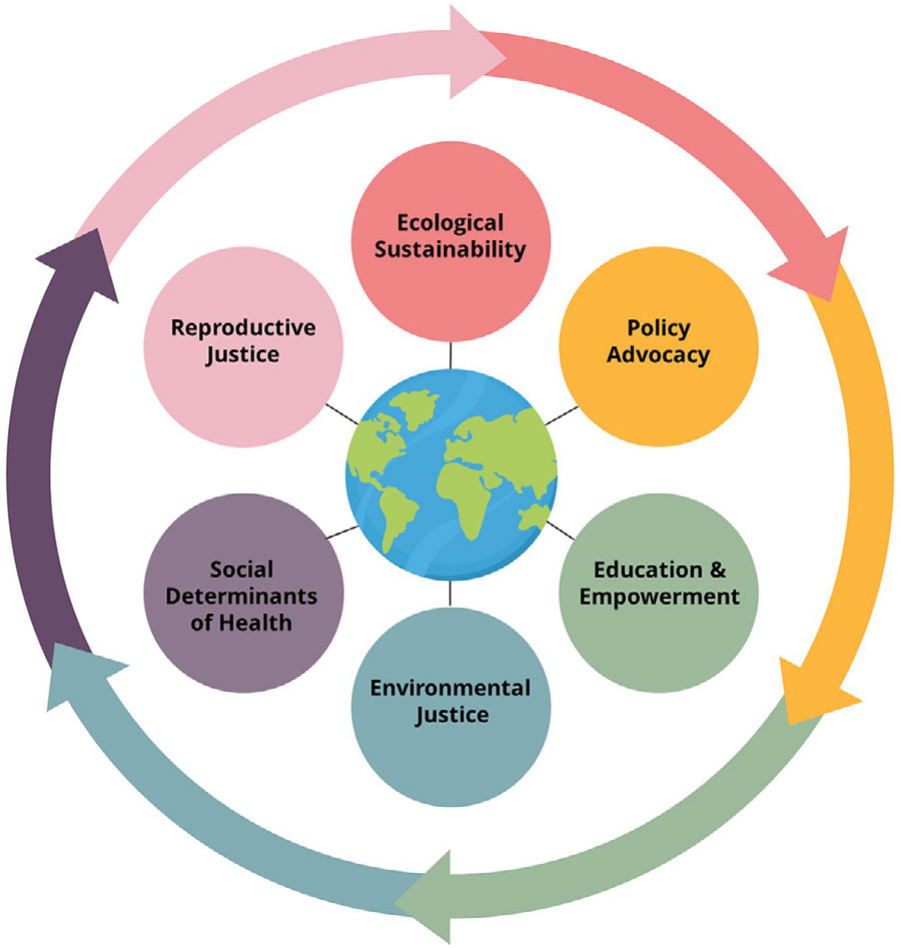
Graphic Model of Midwifery's Role in Planetary Health A visual representation of the 6 interconnected domains of midwifery care, demonstrating how integrating sustainability, social justice, and ecological awareness positions midwives as essential partners in practice transformation, education, and advocacy for a healthier planet.

The 6 domains function as an interconnected system reflecting planetary health and recognizing that healthy pregnancies and communities depend on a healthy planet and that achieving this requires coordinated action across policy, sustainability, justice, social determinants, and education rather than isolated efforts.

## MIDWIFERY'S ROLE IN PLANETARY HEALTH TOOLKIT: IMPLICATIONS FOR EDUCATION, POLICY, AND PRACTICE

Midwives, grounded in holistic and community‐centered care, have a critical role to play in advancing planetary health and addressing the complex challenges posed by environmental change.^25^ Integrating planetary health principles into midwifery education, policy, and practice offers a significant opportunity to enhance care quality while promoting sustainability and equity.^23^, ^26^ The Graphic Model of Midwifery's Role in Planetary Health serves as a foundational framework to guide midwives in embracing their multifaceted role as advocates for maternal health, environmental stewardship, reproductive justice, and social equity. Ongoing research, education reform, and policy development will be essential to fully realize the potential of midwifery as a driver of planetary health and resilient communities.^27^, ^28^

To aid midwifery educators and clinicians with integration of this graphic model into the various areas in which midwives work, a toolkit has been developed by the author, noting focus areas of practice, associated domains of the graphic model, and actionable strategies for integration (see Table 3). By adopting this holistic, systems‐oriented approach, midwifery practice, education, and policy can more effectively address the interconnected social, economic, and environmental determinants that impact maternal and planetary health.

**Table 3:** Planetary Health Toolkit: Actionable Strategies Across Midwifery Education, Practice, and Advocacy

Focus Area	Associated Domains	Actionable Strategies
Education	Education and empowerment Social determinants of health	1. Integrate climate‐informed case studies and clinical simulations (eg, health impacts of extreme heat exposure, climate‐induced displacement). 2. Incorporate “eco‐audits” into clinical training to identify and implement low‐waste care opportunities. 3. Use interprofessional learning opportunities to collaboratively address environmental and public health crises.
Clinical practice	Ecological sustainability Reproductive justice Environmental justice	1. Include assessments of environmental exposures (eg, air quality, water safety, extreme heat) in routine prenatal and postpartum clinical assessments. 2. Promote low‐intervention, low‐carbon care models (eg, supporting physiologic birth, promoting breastfeeding or chestfeeding). 3. Support clinic‐level sustainability initiatives and broader community climate resilience efforts.
Research and advocacy	Policy advocacy Environmental and social justice	1. Advance research and quality improvement projects examining environmental impacts on perinatal care (eg, correlations between heat and preterm birth, air pollution and hypertensive disorders, or carbon footprints across distinct birth settings). 2. Examine and document systemic inequities in environmental exposures and perinatal outcomes (eg, the impacts of substandard housing, access to green space, and geographic climate vulnerability). 3. Engage actively in policy efforts (eg, developing maternal health climate adaptation plans, advocating for clean air and water protections, and establishing sustainability standards in maternity care).

## CONCLUSION

### Holistic Nature of Midwives

For centuries, midwives have had a deep respect for the cycles of nature and human resilience. Modern midwifery practice across the globe has evolved from spiritual and nature‐based traditions into a culturally significant practice that reflects the unique birthing customs and environmental realities of each society.^29^ Midwifery has long been grounded in an understanding of the natural rhythms of the body and the environment, reflecting the profession's enduring commitment to holistic and person‐centered care.

### Synthesis and Systems Application

The Graphic Model of Midwifery's Role in Planetary Health serves as both a conceptual anchor and a practical toolkit for curricular integration, clinical innovation, and policy development. By operationalizing planetary health principles across multiple levels of midwifery, the model transforms abstract ecological concepts into tangible professional actions. It illustrates the synergy between some of midwifery's core pillars: policy advocacy, ecological sustainability, reproductive justice, social determinants of health, environmental justice, and education and empowerment. Moreover, the model highlights how these dimensions collectively support healthier people and a healthier planet. This model provides a practical and adaptable tool to guide midwifery practice in responding to the evolving challenges of environmental change while centering justice and equity in care delivery.

## CONFLICT OF INTEREST

The author declares no conflicts of interest.
